# Application of 3D printing individualized guide plates in percutaneous needle biopsy of acetabular tumors

**DOI:** 10.3389/fgene.2022.955643

**Published:** 2022-07-22

**Authors:** Wen Wu, Siyu Liu, Lei Wang, Bing Wu, Lulu Zhao, Wenbo Jiang, Kerong Dai, Yongqiang Hao, Lingjie Fu, Songtao Ai

**Affiliations:** ^1^ Department of Orthopaedics, Shanghai Ninth People’s Hospital, Shanghai Jiao Tong University School of Medicine, Shanghai Key Laboratory of Orthopaedic Implants, Shanghai, China; ^2^ Department of Radiology, Shanghai Ninth People’s Hospital, Shanghai Jiao Tong University School of Medicine, Shanghai, China; ^3^ Engineering Research Center of Digital Medicine and Clinical Translation, Ministry of Education, Shanghai, China

**Keywords:** acetabular tumors, individualized guide plate, needle biopsy, 3D printing technology, image registration, and fusion

## Abstract

**Objective:** The objective of the study was to investigate the effectiveness of applying the individualized guide plate which is based on digital image processing and 3D printing technology to percutaneous needle biopsy of periacetabular tumor.

**Methods:** From July 2017 to August 2019, 11 patients (5 males and 6 females, aged 13–70 years, mean 42.3 years) with acetabular tumors diagnosed by needle biopsy in our hospital were enrolled in this retrospective study. Preoperative CT and MRI enhancement examination were performed routinely, and the DICOM data were collected and imported into Medraw Print software. According to the specific anatomical morphology of acetabula, this study adopted the reverse calculation and direct design to print the individualized puncture guide plate using 3D printing technology. The puncture point and sampling approaches were determined by the guide plate morphology and the “double guide-hole and slideable groove” design. First, we evaluated the fitness of the 3D guide plate to the local anatomical structure, its assisted-puncture accuracy was estimated by imaging examinations, and postoperative complications were recorded. The accuracy of the needle biopsy pathological result was estimated with reference to that of the tumor resection.

**Results:** Our results showed that the 3D printing individualized guide plate matched the patients’ pelvic skin well, the puncture approach was consistent with the preoperative design, and no significant anatomical injuries including vascular and neural complications occurred after surgery. Nine patients’ (90%) biopsy results were consistent with their postoperative pathological results, and one patient gave up the tumor resection.

**Conclusion:** Based on digital image processing and 3D printing technology, the individualized guide plate can be used to guide the needle biopsy of acetabular tumors which makes the operation simpler and more precise.

## Introduction

Minimally invasive biopsy is an important part of bone tumor diagnosis and treatment, and it can provide histopathological and grade information and help to optimize the treatment plan (Filippiadis et al., 2018). The acetabulum is the deepest part of the pelvis and also the site most frequently involved in pelvic tumors. However, it is both a challenge and a potential risk to determine the pathological type of bone tumors by few biopsy samples (Layfield et al., 2014). Now, the traditional puncture biopsy technique mostly relies on the surgeons’ experience and the guidance of imaging equipment. The operation is often blind to some extent, which prolongs the operation time and increases the radiation dose and surgical complications (Mitsuyoshi et al., 2006). Because the acetabulum is deep and adjacent to some great blood vessels and nerves, minimally invasive puncture is difficult to take samples accurately. Therefore, the preoperative plan should be formulated in detail. With the rise of 3D printing technology, individualized guide plates have been applied to many orthopedic disorders, like trauma, spine diseases, joint disease, and bone tumor (Skelley et al., 2019), which provides a theoretical and practical basis for its application in tumor biopsy. This study retrospectively analyzed the clinical data of 11 acetabular tumor patients that performed needle biopsy in our hospital and preliminarily investigated the feasibility of applying the individualized guide plate to needle biopsy of acetabular tumor, which was based on digital image processing and 3D printing. The method introduced in this study can also be applied to radiofrequency ablation and bone cement perfusion of acetabular metastatic tumors.

## Materials and methods

### Clinical cases data

Inclusion criteria: from July 2017 to August 2019, patients admitted to our hospital diagnosed with acetabular tumors by clinical history and imaging examinations were included, and the tumors’ pathology was uncertain unless the intraoperative biopsy was performed. Preoperative CT and MRI enhanced examination of the lesion area were performed. There were no surgical contraindications. Imaging examinations suggested the tumor location was deep. The tumor was too heterogeneous to obtain samples by routine biopsy.

Exclusion criteria: patients with imaging examination contraindications or did not perform the individualized guide plate-assisted biopsy were excluded. All patients went through a one-month design cycle that includes admission, refinement of tests, design of the surgical plan, printing of the guide, and final surgery. This study was approved by the Ethics Committee of Shanghai Ninth People’s Hospital, Shanghai Jiao Tong University School of Medicine.

### Image examination and assessment

The CT scans were performed using a 128-slice dual-source CT scanner (SOMATOM Definition Flash, SIEMENS, Germany), and different scan sequences were selected according to bone tumor positions. The scanning parameters were set routinely as follows: tube voltage 120 kV, reference current time 333.0 mA s, detector collimation 32 mm × 0.6 mm, pitch 0.6 mm, and slice thickness 0.625 mm, without interval.

The MRI scans were performed using a 3.0T MR scanner (MAGNETOM Verio 3.0T, SIEMENS, Germany). The routine scanning parameters took the enhanced T1WI sequence as the main reference standard: TR/TE = 600/11 ms, FOV adjusted according to the scanning position, and slice thickness 2∼3 mm.

The CT and MRI images of all cases were double-blind estimated by two senior radiologists with 10 years or more of diagnostic imaging experience, and the location, size, and surrounding anatomical structure of the tumors were recorded. If they have a different assessment, they will discuss and decide. All data were stored in DICOM3.0 standard format and imported into medical surgery design software Medraw (YinWei Medical Technology (Shanghai) Co., Ltd. China).

### Design and processing of individualized percutaneous guide plates

**FIGURE 1:**
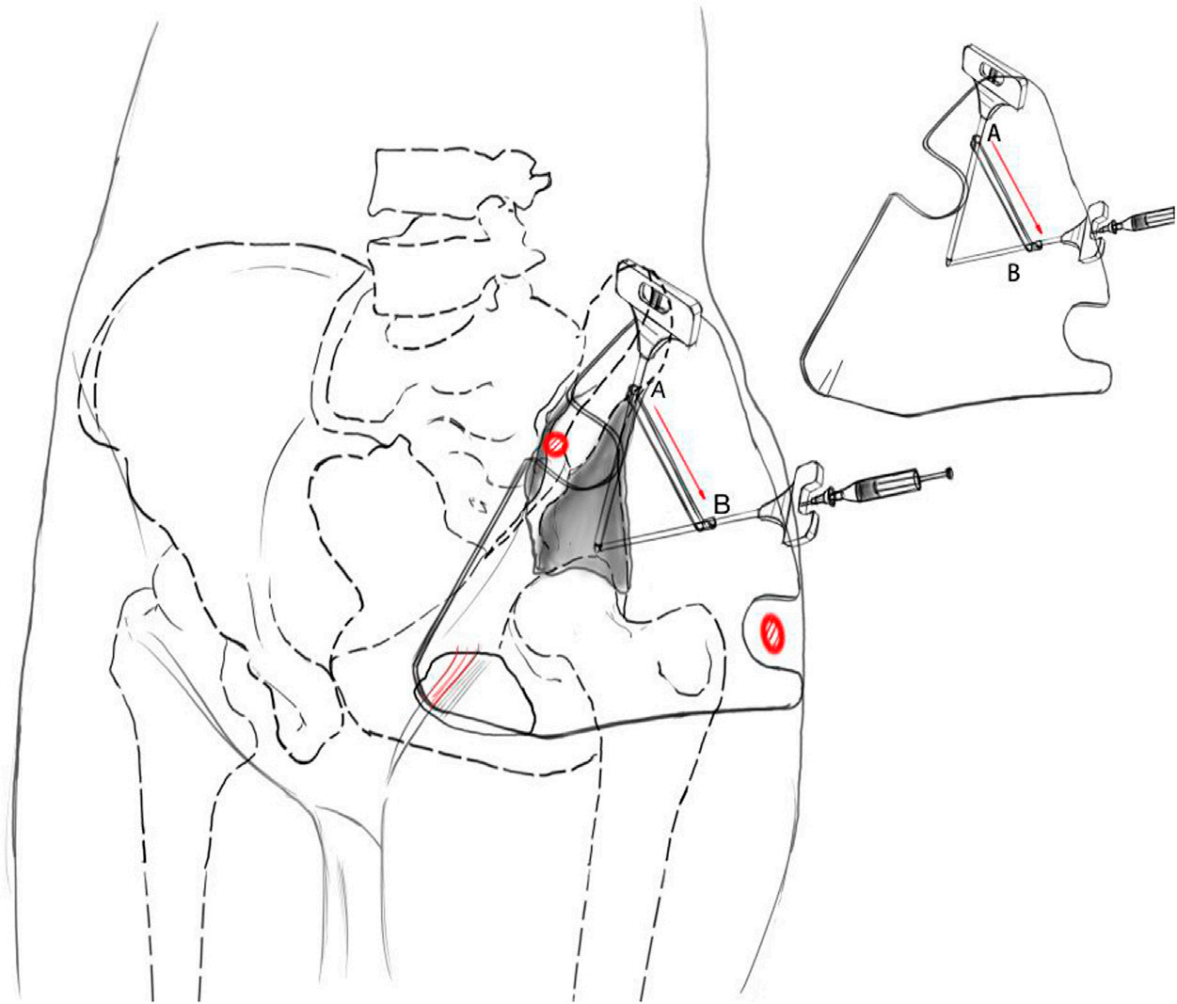
Design concept of the individualized guide plate for 3D printing.

The individualized guide plate adopted some unique designs, including its morphology and guide holes (Figure 1). Its whole morphology was determined by the skin fold morphology of the partial hip joint and inguinal region and by two bony anatomical marks of the pelvis: the greater trochanter of the femur and the anterior superior iliac spine. The guide hole adopted the “double guide-hole and slideable groove design, including the puncture hole A, hole B, and the sampling hole band connecting two holes. Hole A ensured that the needle was located in the ilioinguinal approach of subsequent surgery to avoid great blood vessels and nerves. Hole B ensured the optimal sampling angle of the intraosseous tumors to avoid the needle entering the hip joint and obtained enough tissue specimens at the same time.

According to the aforementioned design plan, all patients’ CT and MRI data were imported into Medraw Print software first, and then the registration and fusion modules of CT and MRI images were selected to adjust different images. Clinicians determined the most metabolically active area within the tumor in MRI images and the target area of puncture in CT images. According to the imaging data, we determined the individualized guide plate morphology which was highly consistent with the patients’ local pelvic morphology. The two “notches” of the guide plate matched the greater trochanter of the femur and the anterior superior iliac crest, respectively. By palpating the bony anatomical marks through the “notches,” two marks were located in the notches to fix the guide plate on the pelvic surface. The design of the guide holes accorded to both the angles of puncture and sampling. Its inner diameter was larger than the outer diameter of the needle to ensure the needle pass through.

Three-dimensional printing technology equipment used was light-cured stereoscopic modeling Objet 260 Connex 3 (Stratasys, Israel); 3D printing material used was a photosensitive resin MED 610(Stratasys, Israel).

The process of 3D printing guide plate: first, we established the connection between the computer and 3D printer and prepared a photosensitive resin. Then, we used Medraw Print software to create a guide plate model. The STL files of individualized guide plate models were generated and imported into 3D printing equipment. Finally, we poured the photosensitive resin into the 3D printer and started printing after adjusting the parameters. After printing the guide plate, the supporting material was removed, cleaned, and disinfected edit at low temperature. Then, the inner diameter of the guide hole was checked and recorded and compared with the preoperative design plan; then it was disinfected again, and the equipment was sealed for surgery.

### Surgical application of the individualized guide plate

**FIGURE 2:**
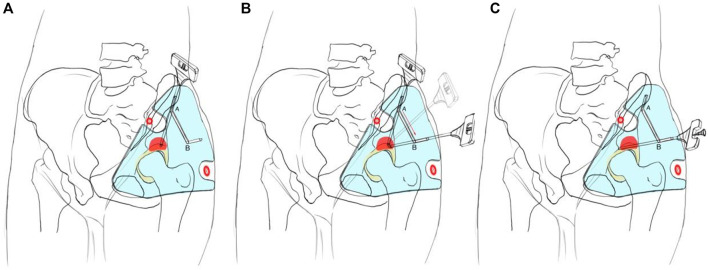
Operation procedure of the 3D printing individualized guide plate.

1) The patient was in a supine position with local anesthesia, and the needle biopsy was performed in the routine procedure (Figure 2). The bony anatomical marks were determined by palpation, including the anterior superior iliac spine, the iliac crest, and the greater trochanter of the femur. According to the anatomical characteristics of the groin skin morphology, we fixed the guide plate and checked its match degree.
2) The puncture point was located in the ilioinguinal approach of subsequent surgery, about 1 cm behind the anterior superior iliac spine on the lateral iliac crest. The puncture angle was parallel and close to the iliac outer plate (Figure 2A). The puncture point and its puncture angle were consistent with those of guide hole A. A biopsy needle was selected (8G, TSK, Japan) to reach the acetabular cortex through the skin and guide hole A.
3) We kept the needle tip against the acetabular cortex and adjusted the angle so that it could slide from guide hole A to guide hole B along the linear groove, and its angle was finally consistent with that of guide hole B (Figure 2B).
4) The hollow sleeve was replaced. The needle was inserted into the target area of the bone tumor along the guide hole B angle, and the puncture depth was measured preoperatively for sampling (Figure 2C). Intraoperative X-ray fluoroscopy was performed to verify the needle’s location, and the mark range was consistent with the preoperative design. Samples were taken and sent for pathological examination. The wound was closed layer by layer and bandaged with an aseptic dressing.

Postoperative complications were recorded such as local hematoma formation, infection, and vascular and nerve injury. The pathological results of needle biopsy were compared with those of the tumor resections.

## Results

**FIGURE 3:**
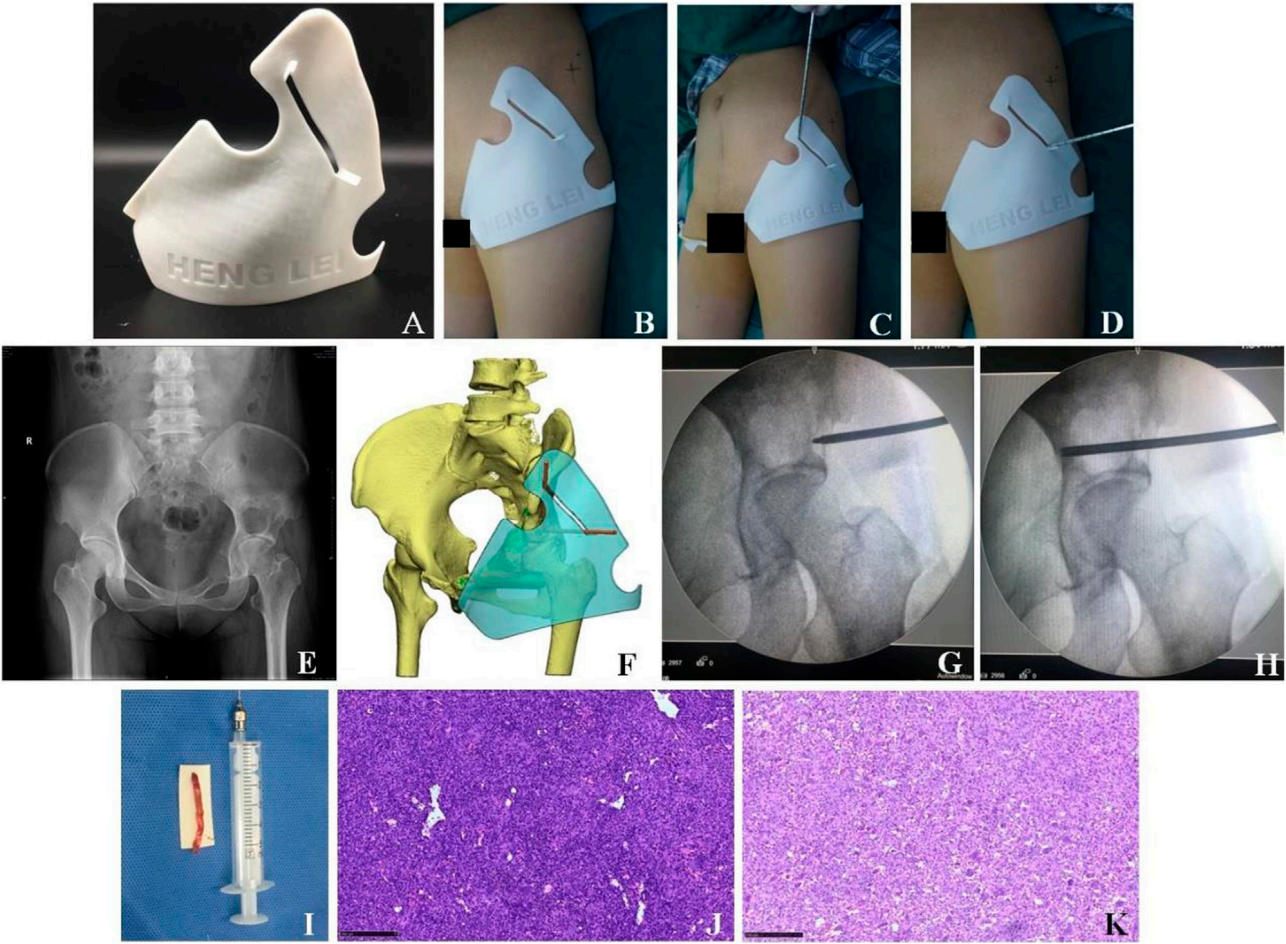
Clinical treatment of the 3D printing individualized guide plate. **(A)** Appearance of the 3D printing individualized guide plate. **(B–D)** Surgical procedures. **(E)** X-ray showed the bone tumor in the left acetabulum. **(F)** 3D model of the individualized guide plate. **(G–H)** Puncture location was confirmed by intraoperative X-ray fluoroscopy. **(I)** Tissue sample was obtained by needle biopsy. **(J)** Pathological section was obtained by needle biopsy (×100, HE staining). **(K)** Pathological section (×100, HE staining) after the tumor resection suggested giant cell tumor of bone, which was consistent with that of **(J)**.

This study enrolled 11 cases of periacetabular bone tumor in total, including 5 males and 6 females, aged from 13 to 70 years, with an average age of 42 years. All patients’ individualized guide plates basically matched the anatomical structure of puncture sites, and their puncture angles and depth were consistent with the preoperative plans. All patients were in good condition intraoperatively and did not show any abnormalities. No vascular and neural injuries occurred after surgery. We selected a case of acetabular bone giant cell tumor for further discussion (Figure 3). In addition, one patient was diagnosed as low-grade malignant tumor by biopsy pathology and his family finally gave up the surgical treatment due to economic factors. The biopsy results of 9 patients (90%) were consistent with their own postoperative pathological results (Table 1).

**TABLE 1:** Basic clinical data of patients with acetabular tumor.

Number	Gender	Age	Location	Biopsy result	Postoperative pathological examination result	Postoperative complication
1	Male	70	L	Abundant osteoid tissue with few heteromorphic tumor cells, consistent with osteosarcoma	Osteosarcoma with partially sclerosing	—
2	Male	54	L	Metastatic bone tumor	Bone metastasis of thyroid papillary carcinoma	—
3	Male	42	L	Tends to be fibro-osseous lesions	Fibrous dysplasia with abundant spindle cells in some stroma	—
4	Female	53	L	Malignant pelvic tumor	Osteosarcoma with local degeneration and necrosis	—
5	Female	33	R	Spindle cell tumor, with multinucleated giant cells, tended to be giant cell tumor of bone with fibrous histiocytoma-like hyperplasia	Aggressive giant cell tumor of bone combined with an aneurysmal bone cyst	—
6	Female	51	L	Most are denatured/cellulose-like exudates without obvious tumor	Pigmented villonodular synovitis (diffuse giant cell tumor of the tendon sheath) and abundant cells with atypical hyperplasia, consistent with malignant change	—
7	Female	22	R	Giant cell tumor of bone	Invasive giant cell tumor and some stromal cells with atypia, with a focal aneurysmal bone cyst	—
8	Male	40	L	Small cell malignant tumors with hemangiopericytoma-like changes, tended to be mesenchymal tumors	Mesenchymal chondrosarcoma	—
9	Male	54	L	Chondrosarcoma with few cartilage tissues and atypia cells	Dedifferentiated chondrosarcoma of the left pelvis	—
10	Female	13	L	Spindle cell tumor with some atypia cells, low-grade malignant tumor	—	—
11	Female	33	L	Giant cell lesion/tumor with abundant spindle cell and partial collagen degeneration	Giant cell tumor of bone with partial active cell proliferation	—

Note: “—” means that patient did not do bone tumor resection.

## Discussion

Bone tumor biopsy usually uses imaging equipment to determine the puncture point of the lesions. Because the needle biopsy under X-ray fluoroscopy hardly provides three-dimensional spatial information of the lesions, it fails to accurately locate the bone tumor when only part of the bone cross section is involved. CT-assisted needle biopsy is often applied to small, sclerosing, cystic fluid, deep lesions such as in vertebrae and pelvis or lesions adjacent to vascular and nerve bundles (Hryhorczuk and Biermann, 2011; Chang et al., 2015). Its defects include the following: 1) the large radiation dose; 2) the tissue resolution is too low to determine the tumor active area; 3) limitation of the coil of CT equipment is that the puncture operation is stiff in fat patients or in some areas such as the pelvis. MRI has the advantages of high soft-tissue contrast, multi-plane imaging, accurate positioning, selective access approach, and no radiation. However, MRI-assisted biopsy of bone and soft tissue systems requires corresponding equipment (Ojala et al., 2002; Weiss et al., 2008), which limits its clinical application. Other imaging techniques have also been reported, including combining ^18^F-deoxyglucose positron tomography (FDG-PET) with MRI to guide biopsy to locate the most active and malignant area of the tumor (Hain et al., 2003; Sheikhbahaei et al., 2015). In addition, using a gamma probe to locate the rib lesions by single-photon emission CT (ECT) during operation can reduce the operation time and avoid excessive resection (Sodha et al., 2004). But now these new biopsy techniques also require specific imaging equipment, so it is difficult to get a clinical promotion.

Because the acetabulum is deep in the pelvis and the tumor morphology of this area is irregular, needle biopsy is often difficult. The increasing 3D printing technology can provide highly individualized treatment for this kind of patients (Zhou et al., 2014). The digital surgery plan can be realized by the 3D printing guide plate (Yan et al., 2016; Guan et al., 2017). The guide plate design and preoperative plan can be completed at the same time. We design its contact surface by the hip joint morphology and specific bone anatomical marks, and the guide hole with a fixed angle can accurately guide the needle. The processed guide plate can realize the preoperative plan and guide the surgeon to perform (Omori et al., 2014). By using Medraw Print digital design software, this study integrates the information reflecting the most active area of bone tumor in MRI images into CT images in CT and MRI image registration and fusion module, so as to determine the sampling sites and improve the accuracy of needle biopsy.

The novel guide plate design is the core of this study. We have accumulated some experience through the discussion with engineers and clinical practice (Yang et al., 2018). During designing the individualized guide plate, attention should be paid to the following aspects:

1) The basis of puncture is the guide plate located accurately on the skin surface, and one can realize it by combining the “soft” marks with “hard” ones. The “soft” means the guide plate morphology in the transition area, from the hip joint to the lower abdomen. It is determined by that of the local soft tissue in the hip joint and groin area. The “hard” means the bony anatomical marks of the hip joint, of which the most characteristic ones are the greater trochanter of the femur and the anterior superior iliac spine. Both have obvious apophyses during palpation. We found that the thin patients lacked obvious soft tissue features and had flat skin and shallow groin, but their bony marks were often more obvious, while the obese patients’ bony marks were difficult to palpate, and their soft tissue features were more obvious, such as deep skin wrinkles in the groin area. Therefore, we considered comprehensively the skin morphology and bony marks during designing the guide plate, so as to achieve its accurate body surface location. Usually, the well-designed guide plate is highly compatible with the morphology of the lesion area, which reflects the combination of the 3D printing design and processing technology.
2) The highlight of this design is the guide holes on the guide plate. The guide hole was usually single-hole and unidirectional in tradition, but we designed the double guide-hole and slideable groove. Puncture guide hole A ensures that the puncture needle is located in the ilioinguinal approach of the subsequent surgery, and its angle is parallel to the ilium. Sampling hole B ensures the optimal angle of sampling the tumors after the needle breaks through the acetabular bone.

The linear groove ensures that the needle can glide smoothly from hole A to hole B. The needle is first inserted into hole A. Then, it glides from hole A to hole B, and finally takes samples from hole B. The angle between the approach of hole A and that of hole B is often less than or equal to 60°, and the distance between two holes is often less than or equal to 10 cm. Because the skin is elastic, the operation should be performed smoothly through the double guide-hole and chute design. In the needle biopsy of acetabular tumor, if only hole A is designed, the needle will wrongly enter into the hip joint and cause additional injury and potential tumor tissue spread because its insertion angle is toward the hip joint. Because the needle insertion path is not in the subsequent surgical approach, if only hole B is designed, the tumor in the insertion approach cannot be completely resected by surgery. Some studies reported the percutaneous biopsies of acetabular lesions under the guidance of imaging equipment. The puncture points were all along the transgluteal muscle approach, which were close to sampling hole B of this study (Vaishya et al., 2016; Kamath and Kamath, 2019). Although their puncture points were close to the acetabulum and facilitated the sampling, if the subsequent surgery was selected along the ilioinguinal approach, it could hardly puncture the whole lesions and cause tumor spread. Others reported the CT-guided biopsy surgeries for deep pelvic lesions from different puncture approaches, but they mainly introduced the pelvic lesions and cannot provide a reference for bone tumors (Gupta et al., 2003; Gupta et al., 2004). This design is unique in two aspects. First, the puncture point can be selected so that the surgeons can resect the whole biopsy approach in subsequent tumor resection. On the other hand, it adopts the most direct sampling approach, so the obtained lesion tissues are more complete and helpful for the subsequent pathological diagnosis. In addition, in terms of the puncture of acetabular tumors, this individualized guide plate can also be directly applied to the iliac region. Moreover, the design concept of this guide plate can be applied to other bone tumor biopsies that their puncture angle differs from the sampling angles.

No vascular and neural complications occurred in this study, demonstrating that individualized guide plate assisted-puncture is safe. However, it also has some limitations, such as short duration, fewer cases, no randomized controlled trial to further evaluate its accuracy, and slightly soft printed by the resin. Therefore, we should further shorten the guide plate design and production time to meet the clinical needs. In addition, the increasing cases can also enrich statistical studies.

In conclusion, based on the digital design and 3D printing technology, the percutaneous needle biopsy guide plate can perform the individualized tumor biopsy of complex areas such as acetabulum. Simultaneously, it is safe, accurate, and repeatable, which deserves the clinical application. We hope that the digital orthopedic technology will play a more significant role in more fields in the near future.

## Data Availability

The original contributions presented in the study are included in the article/Supplementary Material; further inquiries can be directed to the corresponding authors.
